# Induced mitochondrial membrane potential for modeling solitonic conduction of electrotonic signals

**DOI:** 10.1371/journal.pone.0183677

**Published:** 2017-09-07

**Authors:** R. R. Poznanski, L. A. Cacha, J. Ali, Z. H. Rizvi, P. Yupapin, S. H. Salleh, A. Bandyopadhyay

**Affiliations:** 1 Faculty of Bioscience and Medical Engineering, Universiti Teknologi Malaysia, 81310 Johor Bahru, Malaysia; 2 Laser Centre, Ibnu Sina ISIR, Universiti Teknologi Malaysia, 81310 Johor Bahru, Malaysia; 3 Computational Optics Research Group (CORG), Ton Duc Thang University, District 7, Ho Chi Minh City, Vietnam; 4 Faculty of Electrical & Electronics Engineering, Ton Duc Thang University, District 7, Ho Chi Minh City, Vietnam; 5 Centre for Biomedical Engineering, Universiti Teknologi Malaysia, 81310 Johor Bahru, Johor, Malaysia; 6 Research Center for Advanced Measurement and Characterization, National Institute for Materials Science, Tsukuba, 305-0047 Japan; Southeastern Louisiana University, UNITED STATES

## Abstract

A cable model that includes polarization-induced capacitive current is derived for modeling the solitonic conduction of electrotonic potentials in neuronal branchlets with microstructure containing endoplasmic membranes. A solution of the nonlinear cable equation modified for fissured intracellular medium with a source term representing charge ‘soakage’ is used to show how intracellular capacitive effects of bound electrical charges within mitochondrial membranes can influence electrotonic signals expressed as solitary waves. The elastic collision resulting from a head-on collision of two solitary waves results in localized and non-dispersing electrical solitons created by the nonlinearity of the source term. It has been shown that solitons in neurons with mitochondrial membrane and quasi-electrostatic interactions of charges held by the microstructure (i.e., charge ‘soakage’) have a slower velocity of propagation compared with solitons in neurons with microstructure, but without endoplasmic membranes. When the equilibrium potential is a small deviation from rest, the nonohmic conductance acts as a leaky channel and the solitons are small compared when the equilibrium potential is large and the outer mitochondrial membrane acts as an amplifier, boosting the amplitude of the endogenously generated solitons. These findings demonstrate a functional role of quasi-electrostatic interactions of bound electrical charges held by microstructure for sustaining solitons with robust self-regulation in their amplitude through changes in the mitochondrial membrane equilibrium potential. The implication of our results indicate that a phenomenological description of ionic current can be successfully modeled with displacement current in Maxwell’s equations as a conduction process involving quasi-electrostatic interactions without the inclusion of diffusive current. This is the first study in which solitonic conduction of electrotonic potentials are generated by polarization-induced capacitive current in microstructure and nonohmic mitochondrial membrane current.

## Introduction

The electrophysiological applications of cable theory led Hodgkin and Huxley (H-H) [1] to quantitatively describe voltage-dependent currents obtained by using the voltage-clamp technique. The remarkable success of the H-H model is a mathematical description that relates the microscopic dynamics of gated ion channels to the macroscopic behavior of membrane potential. The H-H equations are foundational because they capture crucial points of analogy between the squid giant axon and in other species both *in vivo* and *in vitro* environments. Although the H-H model portrays the nerve as an electrical analogue in terms of capacitors and conductors, it does not incorporate a physico-chemical understanding of ionic diffusion within the excitable membranes. The Frankenhaeuser and Huxley (F-H) model developed in 1964 [2] was an attempt to include in the H-H model electrodiffusion of ions within the plasma membrane. The F-H model includes electrodiffusion of membrane ion channel permeability based on a description for ionic concentration across membranes where the spatial distance reflects charge spread within the membrane and not within the cytoplasm. Analytical solutions to the F-H equations were obtained when voltage-dependent ionic channels are distributed at discrete positions throughout the membrane based on ionic cable theory [3].

The H-H model is based on electrical cable theory and it would need to be fundamentally revised or replaced if it were based on a physico-chemical footing. This problem is the inability to unify electrodiffusion of ions in electrolytes with cable theory (cf. [4]). Although there were earlier attempts to show electrodiffusive effects on membrane potentials they were fortuitous because of the erroneous equivalence between spatial spread of ionic diffusion and electrical conduction [5]. Since electrodiffusion of ions in an electrolyte applies only at short distances within cellular membranes, therefore a mismatch exists between electrodiffusion models that rely on electrochemical processes based on advection-diffusion equations and electrical conduction that relies on cable equations [6]. Such fortuitous attempts which draw parallel between the electrical representation and electrochemical representation have appeared as ‘molecular models of action potentials’ [7, 8].

Subsequently, there have been more fortuitous attempts at reconciling electrodiffusion models with cable modeling approaches [9–15]. For instance, the diffusive currents have been included in these studies to model electrodiffusion of ions in cylindrical geometries through a single spatial variable that is identical with the conduction of electrical charge in the cable equation. Indeed, the electrodiffusion models based on the classical Nernst-Planck system of equations simply do not provide a description for ionic current flow beyond the width of membranes (nanometers) [6, 16]. In fact, the coupling of cable theory with anomalous electrodiffusion through so-called ‘fractional’ cable equation and ‘fractional’ Nernst-Planck equations [13] can also be fortuitous through attempts at mismatching variables by including separate scaling exponents for both anomalous diffusion across the membrane as in the cystol at the same temporal scale; thereby rendering the approach inadequate for action potentials operating on a much faster time scale in comparison to electrodiffusion of ions.

Despite 60 years of progress [17] still dendritic integration relies on cable theory that excludes microstructure and treats the intracellular medium of neurons as a homogeneous resistive fluid of 70 Ωcm (cf. [18]). However, a resistive fluid is only an approximation to the electrolyte solution. For example, when an ion is attached to a protein-molecule such charged proteins allow for the displacement of ions, where they give rise to polarization-induced capacitive currents. Recent cable models [19] ignore the effects of polarization currents in neurons or include only capacitive effects in the extracellular space [20].

In the same context, cable modeling efforts have included capacitive effects of free charge in the intracellular fluid representative of an electrolytic solution [21]. In this modeling approach, the conduction of free charge of unipolar ions within a passive membrane results in polarization current arising from capacitive charge-equalization and axial capacitive effects. However, the model of Poznanski [21] did not take into account polarization current due to the dispersion of bound charge held by microstructure. An instance where charge dispersal is not ignored, the voltage created by charge ‘soakage’ due to intracellular capacitive effects has been modeled through voltage-dependent capacitors [22, 23].

There are other models that explicitly incorporate voltage-dependent capacitance based on compressive forces acting on the membrane (electrostriction) which are electromechanical in nature (see [24]). In presence of an electric field, changes in membrane thickness due to compressive effects of the electric field are based on the assumption that the membrane bilayer is elastic and can be deformed by an electrostatic force generated by the electric field (electrostriction or electrocompression) resulting in changes to the electrical capacitance of the membrane. Electrostriction is expected to contribute less than 1% of the total capacitance [25] and therefore electromechanical effects can be ignored.

The inclusion of microstructure in the neuronal branchlet is similar, though not identical to electronic analogue, a superconductive ‘neuristor’, with inductor parallel with a resistor component for the intracellular medium (see [26] for a review). However, cable models of neurons include *nonlinear* capacitors instead of inductors and unlike the ‘neuristor’ models, they form a dispersionless system. The microstructure possesses voltage—dependence at slow varying electric fields (e.g., quasi-electrostatic conditions) which enables the capacitor to hold more electric charge than a linear capacitor, resulting in absorption of charge (or charge ‘soakage’) and enhanced electrical signaling. Therefore the polarizibility of the microstructure affects the electrical conduction of electric current through intracellular capacitive effects.

Electrodiffusion models based on the classical Nernst-Planck equations impose a constant-field assumption or the electroneutrality condition [12] rendering it inapplicable for electric potentials within the Debye layer where charge density is neither zero nor constant. For this reason, an alternative route is necessary in terms of a phenomenological description of ionic concentration gradients in an electrolytic microenvironment. One such alternative approach is to modify the cable equation to include the effects of polarized microstructure. This is done by treating the microstructure as a homogenized core-conductor where intracellular capacitive effects arise due to polarization effects of bound charge [23]. The microstructure included polarization-induced capacitive current of charged proteins without endoplasmic membranes [22]. An electrical model of electrolyte solution with endoplasmic membranes in the cytoplasm as a subcellular reticulum cable encased within a core-conductor developed by Shemer et al. [27] did not explicitly take into consideration intracellular capacitive effects due to polarized microstructure.

**Fig 1:**
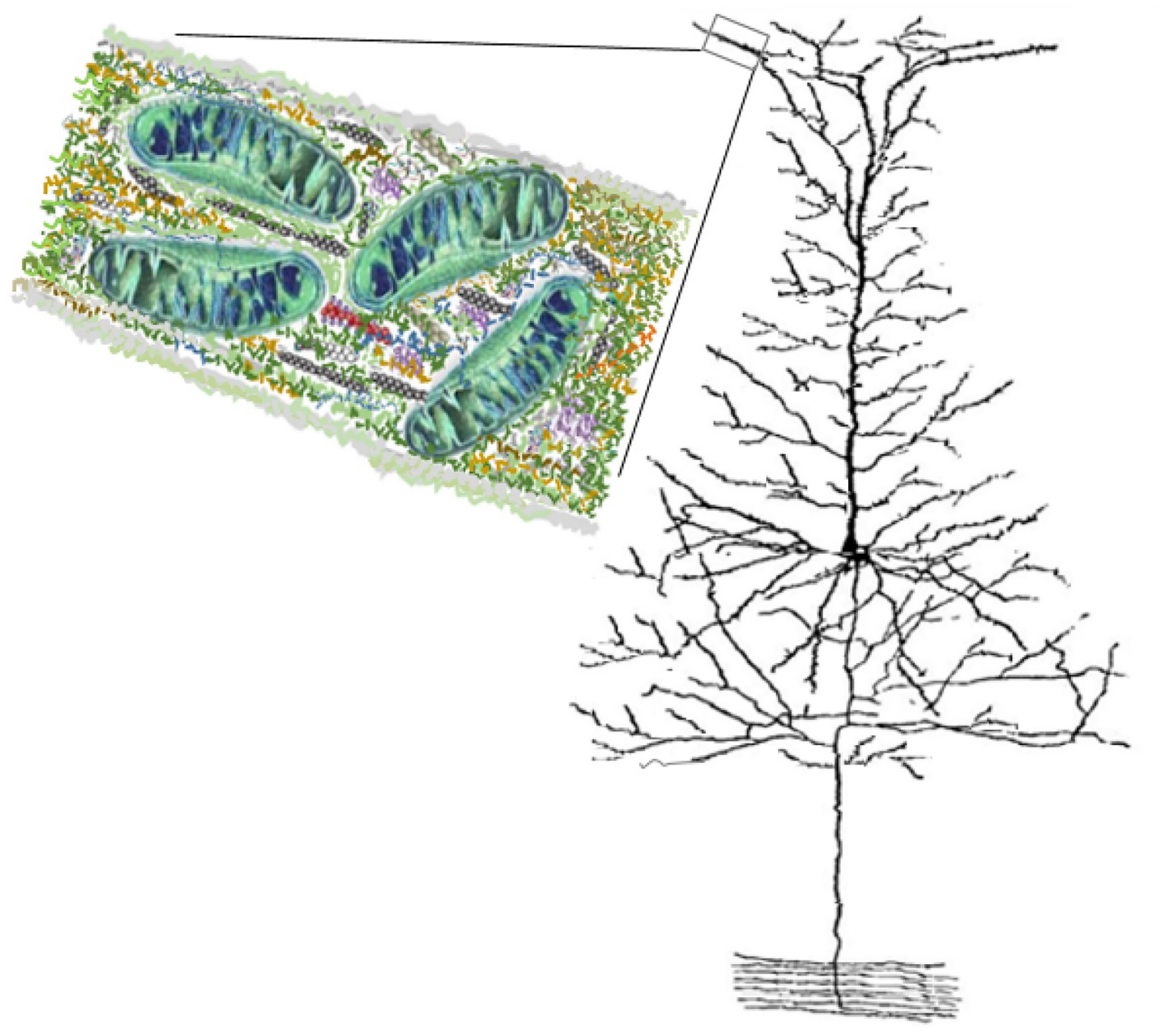
A schematic illustration of the neuronal microstructure. This diagram, drawn more than 110 years ago by Santiago Ramón y Cajal, indicates a pyramidal cell from the cerebral cortex. The inset is a longitudinal section of the neuronal branchlet to illustrate how branchlets are fissured at subcellular scale. The submicron-diameters of the most distal neuronal processes contain a dense meshwork of proteinaceous structures referred to as the microstructure. The microstructure consists of cytoplasm (e.g., water, electrolytes, and polarized free proteins), cytoskeleton (e.g., cytoskeletal bounded proteins, microtubules), and endoplasmic membranes (e.g. mitochondria). Illustrated are mitochondria and the cytoskeleton—interlinking actin filaments, intermediate filaments, and microtubules. The mitochondrion is the largest organelle (∼ 0.2*μm*) within the microstructure and dominates the constituency of the proteinaceous structures since endoplasmic reticulum does not enter into branchlets below a micron.

In this paper, we extend the above approaches by deriving a cable model that considers the effects of changes to ionic concentration gradients through a conduction process, which leads to changes in equilibrium potentials when ions are in solution and ionic flow is inhomogeneous [28]. This is the first study in which electrical conduction of polarization-induced capacitive current in a homogenous core-conductor reflects upon ionic concentration gradients without explicitly modeling electrodiffusion of ions (since cable theory ignores the effects of changes in ionic concentrations that lead to changes in Nernst potentials when molecular ions are in bulk solutions). Consequently, we derive a cable model modified for fissured intracellular medium as illustrated in Fig 1, which includes large organelles like mitochondria in small neuronal branchlets [29].

## Model

The proposed model is not electrochemical as it ignores concentration gradients in electrolyte solutions and lumps all positive and negative charged ions in the cytoplasm (which are not the result of polarization) as free charge. Also included are polarized proteins with electric dipoles that can align to enhance or anti-align to reduce the endogenous electric field caused by the accumulation of bound charge and the bound charge within mitochondrial membranes. Inhomogeneities in the conductivity due to irregular movement of electrical charge in the intracellular fluid of neurons are neglected and displacement current involves the flow of continuous macroscopic charge densities within the Debye layer in the longitudinal direction along the cable.

Cable theory finds its true theoretical foundation in Maxwell’s equations of the electromagnetic field and remains the basis of deriving the cable equation from first principles (i.e., Maxwell’s equations with displacement currents) (see [30]). Application of ∇.*E* = *ρ*/*ε*_0_ in a cylindrical cable of volume (*B*) over a differential distance Δ*x* and radius (*r*) as given in [31]:

$$\begin{array}{c} \frac{1}{\varepsilon_{0}}{\;\int \int \int \;}_{B}\rho \;dv={\;\int \int \int \;}_{B}\nabla .E\;dv=\pi r^{2}\left\{E\left(x+\Delta x,t\right)-E\left(x,t\right)\right\} \end{array}$$ (1)

where *E* is the electric field (*V*/*cm*) assumed to be polarized in the longitudinal direction (along the cable length) *E*(*x*, *t*), *ρ* = *ρ*_*free*_ + *ρ*_*bound*_ are the continuous polarization charge densities in the intracellular medium (*C*/*cm*^3^), *ρ*_*free*_ is the distribution of free charge in positive *x* along the cable (*C*/*cm*^3^) and *ρ*_*bound*_ is bound charge density held by microstrucure in the intracellular space in the positive *x*—direction along the cable (*C*/*cm*^3^).

For an isotropic conductor (cf. [32, 33]), the polarization field *P* in the longitudinal direction (along the cable length) is the electric dipole moment surface density (*C*/*cm*^3^):

$$\begin{array}{c} P=\varepsilon_{0}\left(\varepsilon_{r}-1\right)E \end{array}$$ (2)

where *ε*_*r*_ = 81 is the relative permittivity of water (dimensionless) and fluid permittivity is *ε*_0_ = 7 × 10^−12^(*F*/*cm*), *χ* = (1 − *ε*_*r*_) is the susceptibility of the medium and the term *ε* = *ε*_*r*_*ε*_0_ denotes the permittivity that characterizes the response of the system in terms of separation of charge in the presence of a quasi-electrostatic electric field (*E*), measured as a capacitance = *επ*Δ*x*(*μF*) where Δ*x* is a segment of cable. By the divergence theorem, Gauss’s law for the polarization field can be stated as $\frac{\partial P}{\partial x}=-\rho_{bound}$, then it can be shown upon differentiating Eq (1) with respect to time and multiplying by −*ε*_0_ the following relation is obtained

$$\begin{array}{c} -{\;\int \int \int \;}_{B}\frac{\partial \rho_{free}}{\partial t}\;dv=-\varepsilon_{0}\pi r^{2}\frac{\partial }{\partial t}\left\{E\left(x+\Delta x,t\right)-E\left(x,t\right)\right\}\;-\;\pi r^{2}\frac{\partial }{\partial t}\left\{P\left(x+\Delta x,t\right)-P\left(x,t\right)\right\} \end{array}$$ (3)

The equation of continuity for the charge density (*ρ*) and the current density (*J*) in a volume (*B*) is given as [31]:

$$\begin{array}{c} -{\;\int \int \int \;}_{B}\frac{\partial \rho_{free}}{\partial t}\;dv={\;\int \int \int \;}_{B}\nabla .J\;dv=\pi r^{2}\left\{J\left(x+\Delta x,t\right)-J\left(x,t\right)\right\}+2\pi r\int_{x}^{x+\Delta x}I_{m}\left(x,t\right)\;dx, \end{array}$$ (4)

where the last term is the positive outward membrane current density (*A*/*cm*^2^) and *J*(*x*, *t*) = *J*_*C*_+*J*_*D*_ is the current density flowing along the cable in the *x*-direction (*A*/*cm*^2^). The conductivity current density *J*_*C*_ = *σE* = (Ohm’s law) where the electric conductivity *σ*(*S*/*cm*) is constant, neglects ionic concentration gradients in the electrolytes and the nature of the different ionic species, and therefore represents ionic homogeneity within the microstructure. The electric displacement field is *D* = *ε*_0_*E* + *P* once differentiated with respect to time yields the displacement current density $J_{D}=\varepsilon_{0}\frac{\partial E}{\partial t}+\frac{\partial P}{\partial t}$. Equating Eq (3) to Eq (4) and using mean-value theorem for the single integral in Eq (4) yields

$$\begin{array}{c} -\varepsilon_{0}\pi r^{2}\frac{\partial }{\partial t}\left\{E\left(x+\Delta x,t\right)-E\left(x,t\right)\right\}-\pi r^{2}\frac{\partial }{\partial t}\left\{P\left(x+\Delta x,t\right)-P\left(x,t\right)\right\}\\ =\pi r^{2}\left\{J\left(x+\Delta x,t\right)-J\left(x,t\right)\right\}+2\pi rI_{m}\left(\phi ,t\right)\Delta x,\;x<\phi <x+\Delta x \end{array}$$ (5)

Dividing by Δ*x* and letting Δ*x* → 0 yields

$$\begin{array}{c} -\varepsilon_{0}\pi r^{2}\frac{\partial^{2}E\left(x,t\right)}{\partial t\partial x}+\pi r^{2}\frac{\partial \rho_{bound}\left(x,t\right)}{\partial t}=\pi r^{2}\frac{\partial J(x,t)}{\partial x}+2\pi rI_{m}\left(x,t\right), \end{array}$$ (6)

where *πr*^2^*ρ*_*bound*_(*x*, *t*) = *q*(*x*, *t*) is the surface bound charge per unit length of cable in the positive *x*—direction (*C*/*cm*).

**Fig 2:**
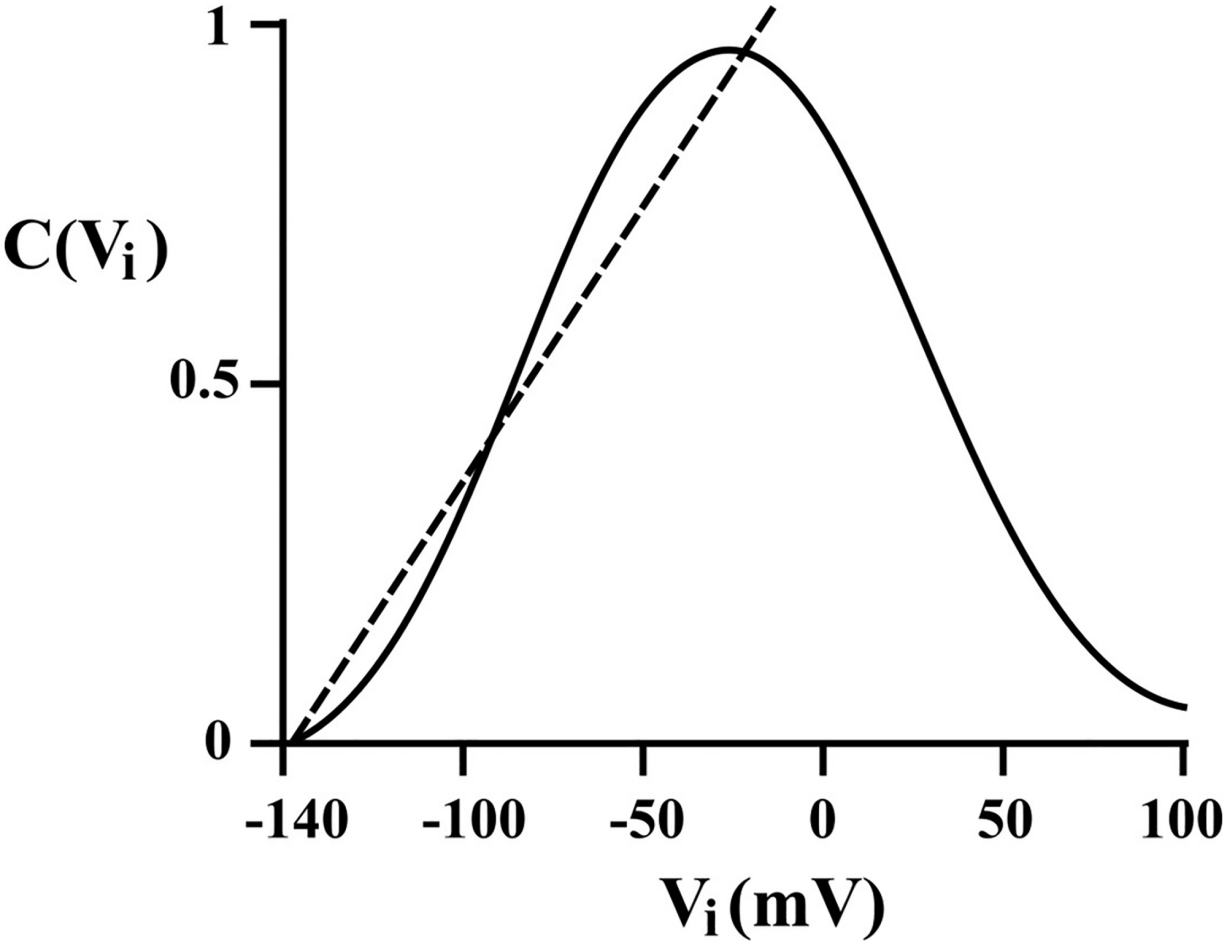
The polarization capacitance-voltage characteristic. A nonlinear capacitance-voltage relationship *C*(*V*_*i*_) taken from the giant squid axon [37] is approximated through a linear polarization capacitance-voltage characteristic as indicated by the dotted line and Eq (7) for typical mitochondrial membrane potential range in cultured rat cortical neurons.

The voltage-dependent charge transfer in the squid axon without microstructure, but due to electrocompression, follows a quadratic dependence (see Fig 2), since the electrostatic force, exerted on a membrane by voltage is given by [34]:

$$\begin{array}{c} \frac{1}{2}C_{m}V^{2}/\Delta_{M} \end{array}$$

where Δ_*M*_ is the membrane thickness. Changes in capacitance due to compressive forces acting on the membrane (electrostriction) are electromechanical. One such example is the voltage-dependent longitudinal (axial) capacitance *C*_*i*_(*V*_*i*_) characterized by a quadratic dependence on the voltage (see [25]):

$$\begin{array}{c} C_{i}\left(V_{i}\right)=C_{i}\left(1+\xi V_{i}^{2}\right) \end{array}$$

where *C*_*i*_ is the voltage-independent longitudinal capacitance (F/cm), *V*_*i*_ is the intracellular membrane potential (mV), and *ξ* is fraction increase in capacitance per square millivolt (*mV*^−2^).

Mitochondrial membrane potential is regulated between −158mV and −108mV [35], which means it is linearly proportional to *V*_*i*_ in the range (see Fig 2). This is the electrical potential range where ion channels from outer mitochondrial membranes are activated. The nonlinear polarization capacitance-voltage characteristic for microstructure can be approximated as a polynomial power in *V*_*i*_, for example $C=2\alpha V_{i}^{0.73}$ [36] or as shown in Fig 2 can be linearized:

$$\begin{array}{c} C\left(V_{i}\right)=2\alpha V_{i} \end{array}$$ (7)

where *α* > 0 is the ‘soakage’ parameter (*mV*^−1^) determined from the electrical charge stored in the capacitance of the microstructure. The parameter represents the capacity to hold more charge or electrical energy than a linear capacitor.

The *nonlinear* capacitor represented by a voltage-dependent longitudinal capacitance *C*_*i*_(*V*_*i*_) = *C*_*i*_*C*(*V*_*i*_) is defined as charge transfer $\frac{dq}{dV_{i}}$ and *C*(*V*_*i*_) is the linearized polarization capacitance-voltage characteristic (dimensionless) generated by the voltage-dependent charge transfer in the microstructure. Upon integration of $\frac{dq}{dV_{i}}$ yields the charge-voltage relationship (electrical charge per unit length of cable *C*/*cm*):

$$\begin{array}{c} Q=C\left(V_{i}\right)V_{i}C_{i} \end{array}$$ (8)

where Q(x, t) = 2q(x, t) is the total surface charge in the microstructure per unit length of cable (C/cm). When *α* = 0 implies there is no charge stored due to the absence of microstructure.

Since the current density is $J=\sigma E+\varepsilon_{0}\frac{\partial E}{\partial t}+\frac{\partial P}{\partial t}$ and under a quasi-electrostatic electric field $E=-\frac{\partial V_{i}}{\partial x}$ it can be shown that

$$\begin{array}{c} \pi r^{2}\frac{\partial J}{\partial x}=\frac{-1}{r_{i}}\frac{\partial^{2}V_{i}}{\partial x^{2}}-\frac{c_{i}}{2}\frac{\partial^{3}V_{i}}{\partial t\partial x^{2}}-\frac{\partial q(x,t)}{\partial t}, \end{array}$$ (9)

where *V*_*i*_ is the intracellular potential, *c*_*i*_ is the axial capacitance across unit length *c*_*i*_ = 2*ε*_0_*πr*^2^(*Fcm*), and *r*_*i*_ is the intracellular resistance per unit length $r_{i}=\frac{1}{\pi r^{2}\sigma }\left(\Omega /cm\right)$. Substituting Eq (9) into Eq (6) and noting that $\frac{\partial^{2}E\left(x,t\right)}{\partial t\partial x}=-\frac{\partial^{3}V_{i}}{\partial t\partial x^{2}}$ such that Eq (6) can be written in the form

$$\begin{array}{c} c_{i}\frac{\partial^{3}V_{i}}{\partial t\partial x^{2}}+2\frac{\partial q(x,t)}{\partial t}+\frac{1}{r_{i}}\frac{\partial^{2}V_{i}}{\partial x^{2}}=2\pi rI_{m}\left(x,t\right) \end{array}$$ (10)

At the center of an infinitely long cable, the total longitudinal current must be equal to longitudinal current flowing in both directions, i.e.$\frac{\partial Q(x,t)}{\partial t}=2\frac{\partial q(x,t)}{\partial t}$ and Eq (10) when multiplied by *r*_*m*_ becomes

$$\begin{array}{c} \frac{r_{m}}{r_{i}}\frac{\partial^{2}V_{i}}{\partial x^{2}}+r_{m}c_{i}\frac{\partial^{3}V_{i}}{\partial t\partial x^{2}}+r_{m}\frac{\partial Q(x,t)}{\partial t}=2\pi rI_{m}\left(x,t\right)r_{m} \end{array}$$ (11)

**Fig 3:**
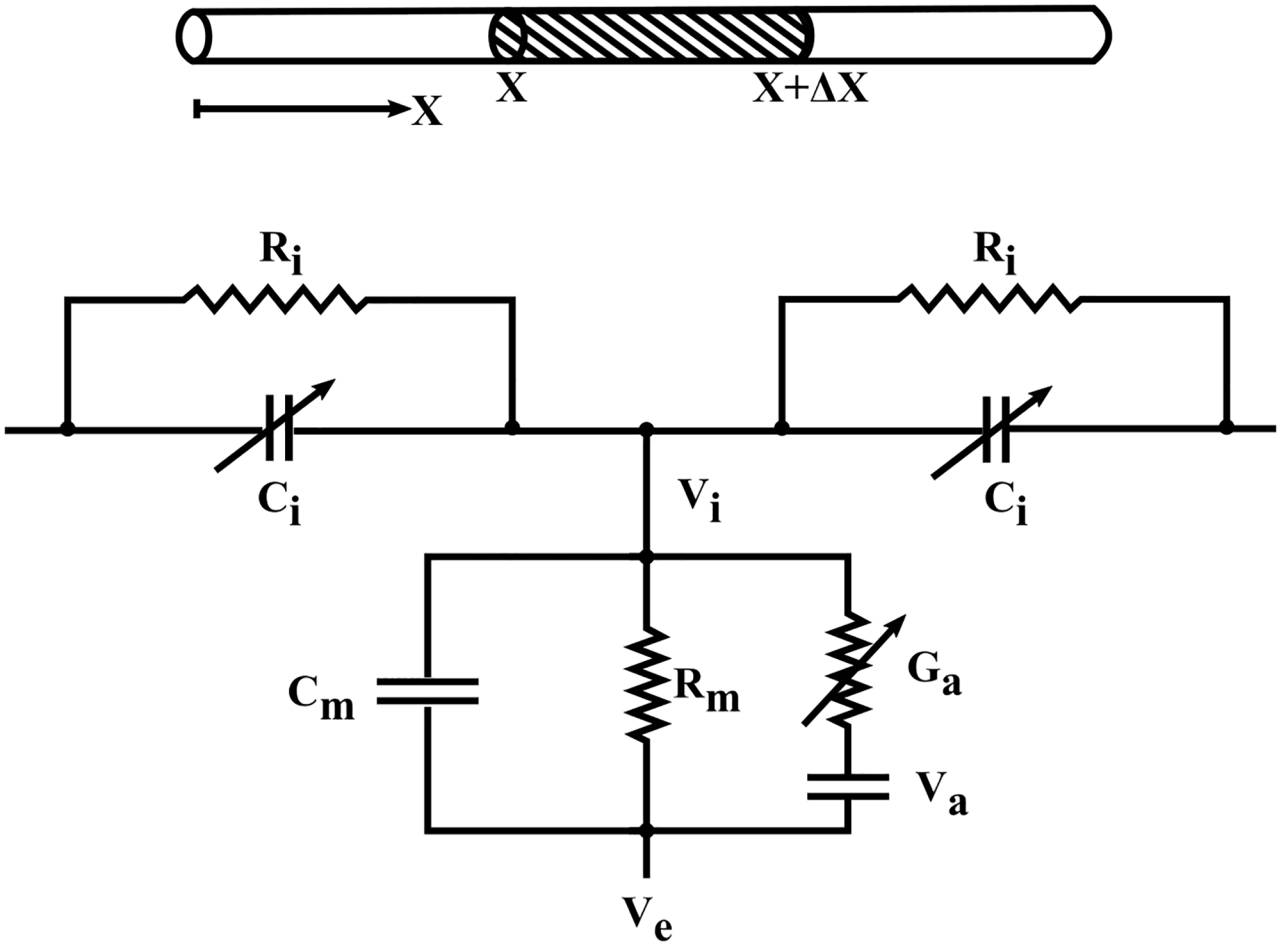
Equivalent circuit of a cable. A cable of small cross-section and infinitely long length that supports electrotonic signals propagating as solitons. The voltage-dependent capacitance originates from a macroscopic phenomenological description of the quasi-electrostatic interactions in the microstructure. It is assumed the cable to be a homogeneous conductor with radial currents ignored and the mitochondrial membrane potential is *V*_*m*_ = *V*_*i*_ − *V*_*e*_ only when *G*_*a*_ ≠ 0; otherwise it represents a passive membrane potential of the neuron without mitochondrial membrane. The length increment (Δ*x*) is shown where arrow indicates the convention that positive charge is in the direction of increasing x, which is the physical distance along the cable. Below is an equivalent series-parallel RC circuit representing a patch of membrane containing both voltage-dependent conductance *G*_*a*_ (mitochondrial membrane) and voltage-independent conductance $G_{m}=\frac{1}{R_{m}}$ in series with the intracellular medium represented by a voltage-dependent longitudinal (axial) capacitance *C*_*i*_(*V*) = *C*_*i*_*C*(*V*_*i*_) of the cable (*F*/*cm*) in parallel with the intracellular resistivity (*R*_*i*_) of the cable (Ω *cm*).

If the conductivity of the extracellular medium is high leaving the extracellular medium isopotential (i.e., *V*_*e*_ = 0) then effect of the external potential on the mitochondrial membrane potential (i.e., *V*_*m*_ = *V*_*i*_ − *V*_*e*_) is negligible. Hence letting *V* = *V*_*m*_ − *E*_*r*_ be the depolarization (*mV*) and *E*_*r*_ be the resting mitochondrial membrane potential (*mV*), together with mitochondrial membrane as shown in Fig 3:

$$\begin{array}{c} i_{m}=2\pi rI_{m}\left(x,t\right)=\frac{V}{r_{m}}+c_{m}\frac{\partial V}{\partial t}+g_{a}\left(V\right)\left(V-V_{rev}\right), \end{array}$$ (12)

where *V*_*rev*_ = *V*_*a*_ − *E*_*r*_ is the reversal potential (*mV*). *V*_*a*_ is the equilibrium potential (*mV*). *i*_*m*_ is the total membrane current per unit length (*A*/*cm*), *I*_*m*_ is the total membrane current density (*A*/*cm*^2^), *g*_*a*_(*V*) is the mitochondrial membrane conductance (*S*/*cm*), *c*_*m*_ is the membrane capacitance per unit length of cable (*F*/*cm*), and *r*_*m*_ is the membrane resistance across a unit length of passive membrane cable (Ω*cm*). Note that *E*_*r*_ = −139*mV* [35], *g*_*a*_(*V*) = 2*πrG*_*a*_(*V*) where *G*_*a*_(*V*) is the mitochondrial membrane conductance per unit area (*S*/*cm*^2^), *R*_*m*_ = 2*πrr*_*m*_ is the membrane resistivity or resistance across a unit area of passive membrane (Ω*cm*^2^), $C_{m}=\frac{c_{m}}{2\pi r}$ is the membrane capacitance per unit area of membrane (*F*/*cm*^2^), $C_{i}=\frac{c_{i}}{\pi r^{2}}$ is the intracellular capacitance per unit length of cable (*F*/*cm*), and *R*_*i*_ = 0.5*σ* is the intracellular resistivity (Ω *cm*). The intracellular resistance per unit length *r*_*i*_(Ω/*cm*) differs from the intracellular resistivity *R*_*i*_ = 0.5*σ*(Ω *cm*) or volume resistivity of the intracellular medium also referred to as specific resistance, which is $\frac{1}{\sigma }$ where *σ* is the electrical conductivity (*S*/*cm*).

Electrical conductivity neglects ionic concentration gradients in the electrolyte solution and the nature of different ionic species and therefore represents ionic homogeneity within the microstructure. However, in our model in addition to bound charge on charged proteins there are inactive membranes encasing the branchlet corresponding to mitochondrial membranes, which also contribute to the displacement current, depending on the equilibrium potential (when the nonohmic conductance is non-zero). The model membrane reverts to a passive neuronal plasma membrane when the mitochondrial conductance is zero. This implies that the mitochondrial membrane channel activity is simultaneous with the opening of plasma membrane channels. This ignores the dependence of the mitochondrial channel on second messengers during synaptic transmission [38].

**Fig 4:**
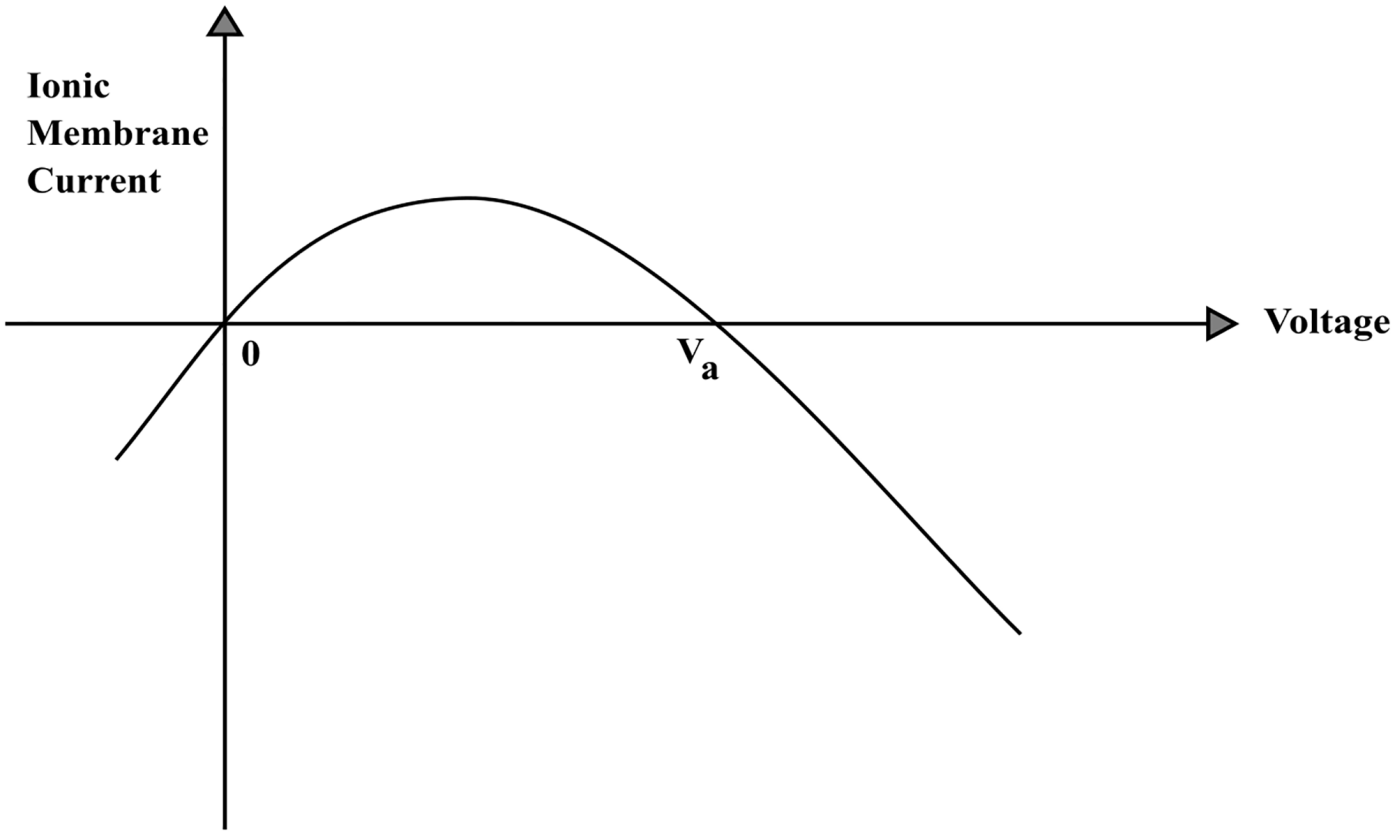
Nonohmicity of charge transfer across the mitochondrial membrane. A theoretically derived charged-carrier model which gives inwardly-rectifying I-V curve of the mitochondrial membrane current per unit length (*A*/*cm*) with the convention that the total ionic membrane current is positive in the outward direction since in this model there is no interstitial space (cf. [27]). The maximum peak occurs at $\frac{a}{4}g_{a}^{*}V_{a}$, when $V=\frac{a}{3b}$. Note that the equilibrium potential $V_{a}=\frac{2}{3}\frac{a}{b}$ is not constant.

The total ionic membrane current per unit length is assumed to be a quadratic nonlinearity as depicted in Fig 4 and represented mathematically by $g_{a}^{*}\left(aV-\frac{3}{2}bV^{2}\right)$. It is based on ions crossing the membrane in combination with charged carrier molecule while the influence of ion concentration gradients are ignored (cf. [39]). The equilibrium potential $V_{a}=\frac{2a}{3b}\left(mV\right)$ and the negative slope conductance is $g_{a}\left(V\right)=-\frac{3}{2}g_{a}^{*}bV$ where the constants are: $g_{a}^{*}$ the maximum conductance of the membrane (*S*/*cm*), ‘a’ (dimensionless) and ‘b’ (*mV*^−1^).

Let *τ*_*m*_ = *c*_*m*_*r*_*m*_ (passive membrane time-constant in msec), $\lambda =\sqrt{\frac{r_{m}}{r_{i}}}$ (electrotonic space-constant in *cm*), Δ = *c*_*i*_*r*_*m*_, from Eq (8): *Q*(*x*, *t*) = *C*(*V*)*VC*_*i*_ is the total surface charge per unit length of cable (*C*/*cm*) and from Eq (7): *C*(*V*) = 2*αV* is a polarization capacitance-voltage characteristic (dimensionless), substituting Eq (12) into Eq (11) yields the so-called nonlinear cable equation:

$$\begin{array}{c} V+r_{m}g_{a}\left(V\right)\left(V-V_{a}\right)+\tau_{m}\frac{\partial V}{\partial t}=\lambda^{2}\frac{\partial^{2}V}{\partial x^{2}}+\Delta \frac{\partial^{3}V}{\partial t\partial x^{2}}+\frac{\Delta }{\pi r^{2}}\frac{\partial \{C(V)V\}}{\partial t} \end{array}$$ (13)

Recasting in terms of dimensionless time $T=\frac{t}{\tau_{m}}$ and space $X=\frac{x}{\lambda }$, and noting the Maxwell time-constant *τ*_*ρ*_ = *c*_*i*_*r*_*i*_ the dimensionless form of the nonlinear cable equation is:

$$\begin{array}{c} V+r_{m}g_{a}\left(V\right)\left(V-V_{a}\right)+\frac{\partial V}{\partial T}=\frac{\partial^{2}V}{\partial X^{2}}+\gamma \frac{\partial^{3}V}{\partial T\partial X^{2}}+\kappa \frac{\partial \{C(V)V\}}{\partial T}, \end{array}$$ (14)

where $\gamma =\frac{\tau_{\rho }}{\tau_{m}}\ll 1$ and $\kappa =\gamma \frac{\lambda^{2}}{\pi r^{2}}$ are both positive constants (dimensionless). The depolarization nondimensionalized via the scaling *U* → *ακV* such that Eq (14) can be written in the form depicting the electrical conduction of electrotonic potentials that propagate as solitary waves under quasi-electrostatic conditions driven by mitochondrial membrane current within polarized microstructure:

$$\begin{array}{c} \left(1+\eta \right)U+\frac{\partial U}{\partial T}-\frac{\partial^{2}U}{\partial X^{2}}=\gamma \frac{\partial^{3}U}{\partial T\partial X^{2}}+2\frac{\partial U^{2}}{\partial T}+\delta U^{2}, \end{array}$$ (15)

where $\eta =ar_{m}g_{a}^{*}$ and $\delta =3g_{a}^{*}b\frac{r_{m}}{2\alpha \kappa }$ are both positive constants (dimensionless). The nonlinear cable equation modified for the inclusion of microstructure is formally a semilinear pseudo-parabolic equation of non-evolutionary type. If *η* = 0 and *δ* = 0 then Eq (15) reduces to a nonlinear cable equation without mitochondrial membrane.

The right-hand side of Eq (15) depicts the intracellular capacitive effects consisting of two terms: (i) the linear dissipative (third-order term) due to charge-equalization, contributes to the longitudinal spread of charge, and (ii) nonlinear terms: one due to charge ‘soakage’ and the other due to the presence of a mitochondrial membrane (absent when *g*_*a*_(*V*) = 0). The third-order term counters the steepness of the voltage gradient due to the nonlinear terms. Given that *γ* is small; the longitudinal polarization current will be conducted with a steep voltage gradient giving grounds for the existence of solitary waves, although, its presence prevents a sharp change in the voltage, so quasi-electrostatic conditions prevail.

## Methods

Nonlinear cable equations may admit solitary wave solutions and if they do they are either severely restricted or approximated [40]. Solitary wave solutions assume a constant conduction velocity that relies on a Galilean transformation of the independent variable which reduces Eq (15) to an ordinary differential equation: *ζ* = (*X* − *X*_*p*_ − *vT*) where *X*_*p*_ is the initial location of the electrotonic signal positioned along the cable and *v* is its velocity (dimensionless). The electrotonic signal is moving towards *ζ* → ∞. The electrotonic signal moving in the other direction *ζ* → −∞ we would use *ζ* = (*X* − *X*_*p*_ + *vT*). For convenience, we use the ansatz U*(*X*, *T*) = Ω(*ζ*) where U* is the free-space version of *U* on an infinite interval (−∞, ∞), with the following identities:

$$\begin{array}{c} \frac{\partial U^{*}}{\partial T}=-v\frac{d\Omega }{d\zeta },\;\frac{\partial^{2}U^{*}}{\partial X^{2}}=\frac{d^{2}\Omega }{d\zeta^{2}},\;\frac{\partial^{3}U^{*}}{\partial T\partial X^{2}}=-v\frac{d^{3}\Omega }{d\zeta^{3}},\;\text{and}\;\frac{\partial U^{*2}}{\partial T}=-v\frac{d\Omega^{2}}{d\zeta }=-2v\Omega \left(\frac{d\Omega }{d\zeta }\right) \end{array}$$ (16)

Substitution of Eq (16) into Eq (15) yields

$$\begin{array}{c} v\gamma \frac{d^{3}\Omega }{d\zeta^{3}}-\frac{d^{2}\Omega }{d\zeta^{2}}+v\left(4\Omega -1\right)\left(\frac{d\Omega }{d\zeta }\right)+\left(1+\eta \right)\Omega -\delta \Omega^{2}=0 \end{array}$$ (17)

with the boundary conditions for electrotonic signals Ω(±∞) = 0.

We obtain solitary wave solutions in free-space using the tanh-function expansion method by introducing a new independent variable [41]:

$$\begin{array}{c} y=tanh(\zeta ) \end{array}$$ (18)

with

$$\begin{array}{c} \frac{d\Omega }{d\zeta }=[\left(1-y^{2}\right)\frac{df}{dy}], \end{array}$$ (19)

$$\begin{array}{c} \frac{d^{2}\Omega }{d\zeta^{2}}=[-2y\left(1-y^{2}\right)\frac{df}{dy}+{\left(1-y^{2}\right)}^{2}\frac{d^{2}f}{dy^{2}}], \end{array}$$ (20)

$$\begin{array}{c} \frac{d^{3}\Omega }{d\zeta^{3}}=[2\left(1-y^{2}\right)\left(3y^{2}-1\right)\frac{df}{dy}-6y{\left(1-y^{2}\right)}^{2}\frac{d^{2}f}{dy^{2}}+{\left(1-y^{2}\right)}^{3}\frac{d^{3}f}{dy^{3}}], \end{array}$$ (21)

where, Ω(*ζ*) → *f*(*y*) and *f*(±1) → 0. Substituting the above new variables into Eq (17) results in the following expression:

$$\begin{array}{c} \left(1+\eta \right)f-v\left(1-y^{2}\right)\frac{df}{dy}=\left[-2y\left(1-y^{2}\right)\frac{df}{dy}+{\left(1-y^{2}\right)}^{2}\frac{d^{2}f}{dy^{2}}\right]-\gamma v[2\left(1-y^{2}\right)\left(3y^{2}-1\right)\frac{df}{dy}\\ -6y{\left(1-y^{2}\right)}^{2}\frac{d^{2}f}{dy^{2}}+{\left(1-y^{2}\right)}^{3}\frac{d^{3}f}{dy^{3}}]-4v\left(1-y^{2}\right)f\frac{df}{dy}+\delta f^{2} \end{array}$$ (22)

The tanh-function expansion method admits the use of a finite expansion of the form $f\left(y\right)=\sum_{n=0}^{n=N}a_{n}y^{n}$ where *n* is a positive integer that will be determined by equating the powers of *y* in the resultant equation upon its substitution into Eq (22). To determine the parameter *n*, we balance the highest-order linear terms with the highest-order of nonlinear terms which gives *n* = 2. Therefore the solution takes the form:

$$\begin{array}{c} f\left(y\right)=a_{0}+a_{0}\left(a_{1}-1\right)y-a_{1}a_{0}y^{2} \end{array}$$ (23)

As *y* → −1 then from Eq (23) and the boundary condition *f*(−1) → 0 yields *a*_1_ = 1 and the solution takes the form:

$$\begin{array}{c} f\left(y\right)=a_{0}\left(1-y^{2}\right) \end{array}$$ (24)

As *y* → 1 then upon substituting Eq (24) into Eq (22) and the boundary condition *f*(1) → 0 yields the dimensionless velocity of the electrotonic signal:

$$\begin{array}{c} v=\frac{1}{2}\frac{3-\eta }{\left(1-4\gamma \right)} \end{array}$$ (25)

where *η* < 3 for positive velocity. Now substitution of *y* = tanh(*ζ*) into Eq (24) yields the traveling wave solution for an electrotonic signal of unitary width and moving at velocity *v*:

$$\begin{array}{c} U^{*}\left(X,X_{p};T\right)=a_{0}sech^{2}\left(X-X_{p}-vT\right) \end{array}$$ (26)

where *a*_0_ > 0 is the dimensionless amplitude determined in S1 Appendix to be $a_{0}\approx 6\frac{1+4\gamma v}{\delta +8v}$ when substituted into Eq (26) together with Eq (25) yields:

$$\begin{array}{c} U^{*}\left(X,X_{p};T\right)\approx 6[\frac{1+2\gamma (1-\eta )}{\delta -4\delta \gamma -4\eta +12}]{\text{sech}}^{2}\left(X-X_{p}-\upsilon T\right) \end{array}$$ (27)

The solitary wave solution governed by Eq (27) is known as a *quasi-soliton* reflecting on the electrotonic signal propagating at a constant velocity *v* > 0 The solitary-wave solution is only an approximate solution of Eq (15) shown in S2 Appendix to be stable based on local stability analysis.

## Results

**Fig 5:**
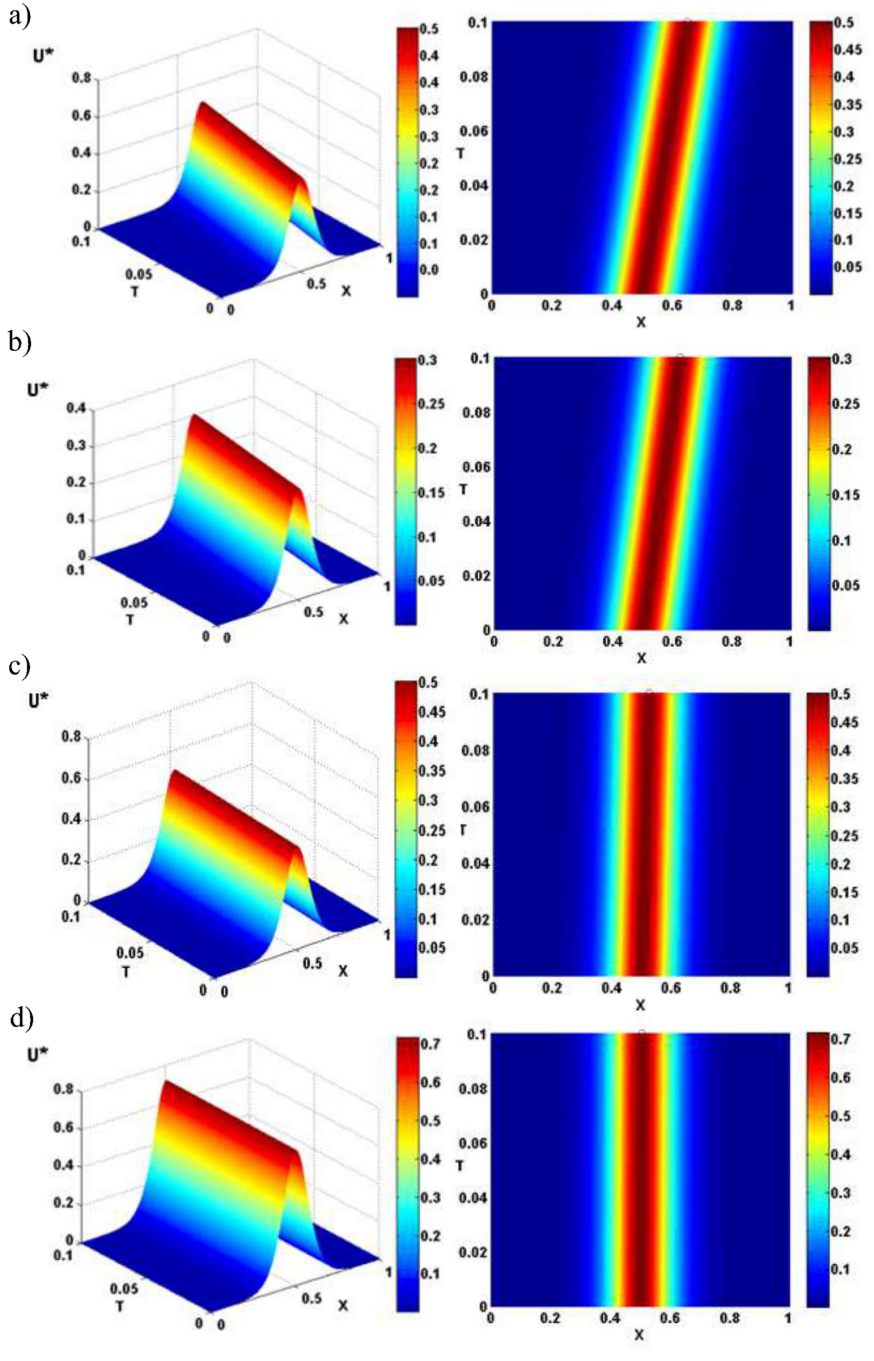
The density plots of quasi-solitons in free-space. Electrotonic signals expressed by a spatiotemporal evolution of free-space voltage (depolarization) U*(*X*, *X*_*p*_;*T*) along an infinite cable of dimensionless distance (*X*) and dimensionless time (*T*). Top-view of the density plot is shown on the right-hand side. The results are presented for a spatially homogeneous medium where the quasi-solitons propagate with a constant velocity and amplitude that is independent of their initial position *X*_*p*_ = 0.5. Electrotonic signals possess no energy loss due to charge ‘soakage’ discharging from the nonlinear capacitor of the polarized microstructure. Parameters used were: (a) *γ* = 0.001, *v* = 1.506, *η* = 0 and *δ* = 0 (passive neuronal plasma membrane), (b) *γ* = 0.001, *v* = 1.255, *η* = 0.5 and *δ* = 10 (mitochondrial membrane), (c) *γ* = 0.001, *v* = 0.251, *η* = 2.5 and *δ* = 10 (mitochondrial membrane), and (d) *γ* = 0.001, *v* = 0.05, *η* = 2.9 and *δ* = 8 (mitochondrial membrane).

The results presented in Fig 5 illustrate the electrotonic signals (or spatiotemporal evolution of depolarization) U*(*X*, *X*_*p*_; *T*) in non-dimensional terms along an infinite cable (in free-space) with passive membrane (Fig 5a) or mitochondrial membrane (Fig 5b, 5c and 5d). The electrotonic signals are insensitive to the initial location of its position along the cable *X*_*p*_ as the hyperbolic secant function reaches a maximum value of unity at *X* = *X*_*p*_. The amplitude of the quasi-soliton is approximately 40% smaller than that for the passive membrane, when the mitochondrial membrane equilibrium potential *V*_*a*_ = 0.2*mV* (cf. Fig 5a and 5b). In this case, the inclusion of the nonohmic conductance simply acts like another ‘leaky’ channel and results in significantly greater current flow through the membrane. However, when the mitochondrial membrane equilibrium potential increases, the peak amplitude of the wave approaches that of the passive neuronal membrane case (cf Fig 5a and 5c). While for *V*_*a*_ = 0.3625*mV* we see that the amplitude of the quasi-soliton becomes greater than the passive membrane case (cf. Fig 5a and 5d). This indicates that the role of the voltage-dependent channels in the mitochondrial membrane is to amplify the quasi-soliton generated by the microstructure. For values of *V*_*a*_ outside the criterion for stability governed by Eq (5) in S2 Appendix are not included.

As shown in Fig 5 (right-hand-side), the velocity of the quasi-soliton is inversely proportional to the slope of this graph. As can be seen the co-ordinate for the first point is fixed at (*X*_1_, *T*_1_) = (0.5, 0). The co-ordinate for the last point is (*X*_2_, *T*_2_) = (0.6506, 0.1) for passive membrane. Thus slope is $=\frac{T_{2}-T_{1}}{X_{2}-X_{1}}=\frac{0.1-0}{0.6506-0.5}=0.664$ and the dimensionless velocity is inversely proportional to this slope 1/0.664 ≈ 1.506 in the passive case. The co-ordinate for the last point is (*X*_2_, *T*_2_) = (0.6255, 0.1) for mitochondrial membrane with *η* = 0.5 and *δ* = 10. The co-ordinate for the last point is (*X*_2_, *T*_2_) = (0.5251, 0.1) for mitochondrial membrane with *η* = 2.5 and *δ* = 10. The co-ordinate for last point is (*X*_2_, *T*_2_) = (0.505, 0.1) for mitochondrial membrane with *η* = 2.9 and *δ* = 8. The slope is $\frac{T_{2}-T_{1}}{X_{2}-X_{1}}=\frac{0.1-0}{0.6255-0.5}=0.7968$ and the dimensionless velocity is inversely proportional to this slope 1/0.7968 ≈ 1.255 in the mitochondrial membrane case with *η* = 0.5 and *δ* = 10. The slope is $\frac{T_{2}-T_{1}}{X_{2}-X_{1}}=\frac{0.1-0}{0.5251-0.5}=3.9841$ and the dimensionless velocity is inversely proportional to this slope 1/3.9841 ≈ 0.251 in the mitochondrial membrane active case with *η* = 2.5 and *δ* = 10. The slope is $\frac{T_{2}-T_{1}}{X_{2}-X_{1}}=\frac{0.1-0}{0.505-0.5}=20$ and the dimensionless velocity is inversely proportional to this slope 1/20 ≈ 0.05 in the mitochondrial membrane case with *η* = 2.9 and *δ* = 8.

If the interaction between two quasi-solitons is robust (i.e. preserves their shape and velocities during the interaction) then quasi-solitons reappear after collision. Amazingly, this is the major property of solitons [42]. Quasi-solitons are dissipative, but only in the sense that in the presence of friction, they gradually decelerate and become smaller and eventually decay as *T* → ∞. In S3 Appendix it is evident for *T* → ∞ that the linearized quasi-soliton dissipates as it propagates. The quasi-soliton is self-generating due to the reservior of electrical charge stored in the capacitance and dissipates only in the absence of microstructure. The *Boussinesq paradigm* which states that the balance between the steepening effect of the nonlinearity and the flattening effect of the dispersion maintains the shape of the soliton [43]. This clearly does not apply to Eq (15) where nonlinearity creates the localized bell-shaped quasi-solitons. Thus in this paper, the term ‘soliton’ is used more generally to refer to a quasi-soliton that asymptotically preserves its shape and velocity on collision with other quasi-solitons [44]. The quasi-solitons are generated by nonlinearity of the charge ‘soakage’ term in Eq (15) and solitonic interaction are the resultant effect of two oppositely directed quasi-solitons admitted from two different Galilean transformations.

Considering the approximate solitonic interactions based on the *summation* of localized traveling waves that can interact without changing their shapes, amplitudes and velocities since linear superposition of quasi-solitons is assumed:

$$\begin{array}{c} U^{*}\left(X,X_{p1}+X_{p2};T\right)=6\frac{1+2\gamma (1-\eta )}{\delta (1-4\gamma )+4(3-\eta )}\left[sech^{2}\left(X-X_{p1}-\upsilon T\right)+sech^{2}\left(X-X_{p2}+\upsilon T\right)\right] \end{array}$$ (28)

**Fig 6:**
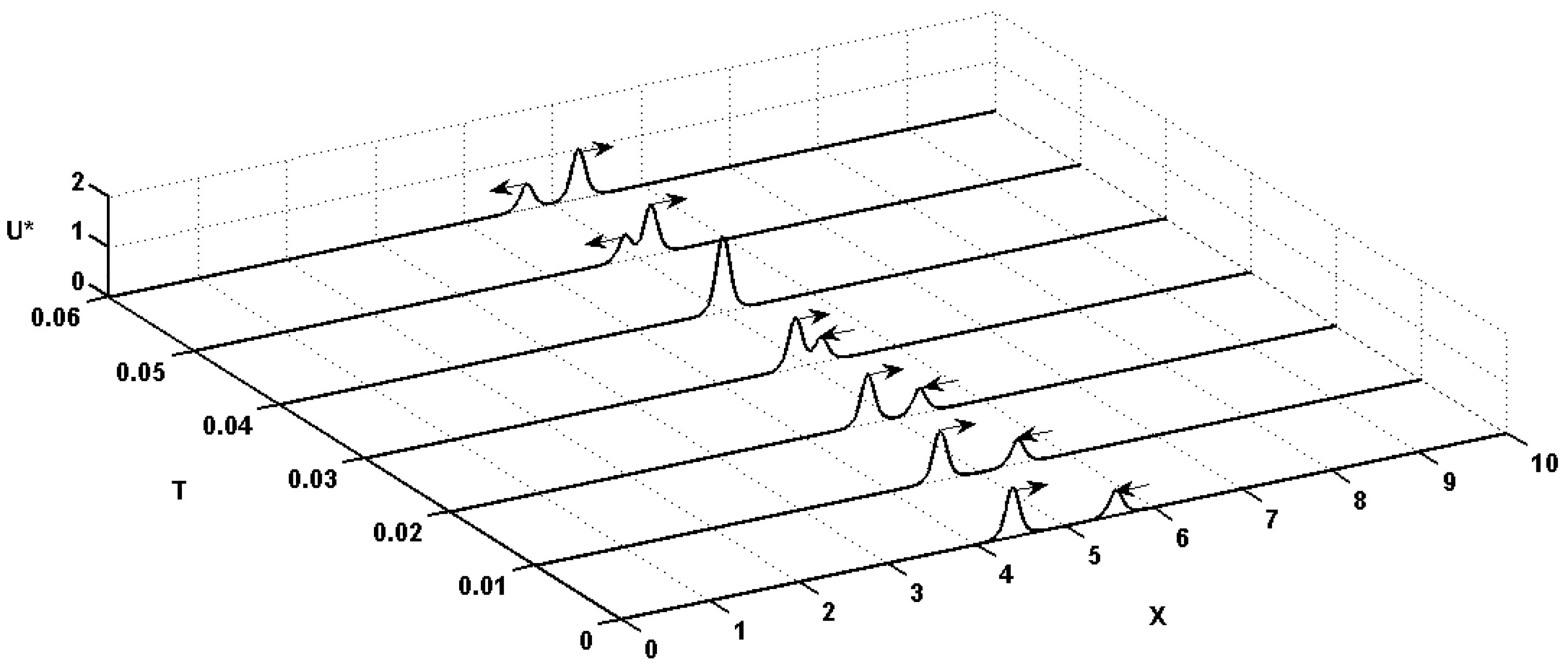
Solitonic interactions between two oppositely directed quasi-solitons. Elastic interaction after a head-on collision along an infinite cable in dimensionless time and space. The electrotonic signals U*(*X*, *X*_*p*_1__ + *X*_*p*_2__;*T*) propagating due to the energy stored in microstructure within neuronal branchlets are obtained from Eq (28) as a function of electrotonic distance (*X*) and dimensionless time (*T*). To differentiate the amplitudes of the electrotonic signals, the signal moving to the right was normalized by U*(*X*_*p*_1__, *X*_*p*_1__ + *X*_*p*_2__; 0) and the signal moving to the left was half-normalized by 2U*(*X*_*p*_2__, *X*_*p*_1__ + *X*_*p*_2__; 0). Both quasi-solitons propagate with a dimensionless conduction velocity of *v* = 1.4558. The elastic interaction between quasi-solitons is taken as a linear superposition. Parameters used were: *γ* = 0.001, *X*_*p*1_ = 4.4177, *X*_*p*2_ = 5.5823, *η* = 0.1, and *δ* = 3 (mitochondrial membrane).

The collision between two quasi-solitons with identical velocities is illustrated in Fig 6. In the case of passive neuronal plasma membrane, the results are similar so will not be reproduced. The elastic collision stems from the absence of a refractory period known to be the cause of collapse between two colliding spikes. The dynamics of interactions (collisions) of the quasi-solitons is elastic (i.e., absorbing one another followed by passing through one another without any change in identity). After linear superposition at collision, the quasi-solitons continue to propagate without dissipating, thus providing unequivocal support for quasi-solitons to be solitons. Likewise, the simulation shows the existence of a point where there is only a single peak, suggesting that the solitons absorb one another during collision. Also the quasi-solitons U*(*X*, *T*) = Ω(*ζ*) are shown to satisfy the following conditions [41]: Ω′(*ζ*) = Ω″(*ζ*) = Ω‴(*ζ*) = 0 where prime denotes differentiation with respect to *ζ*, further reinforcing that quasi-solitons are solitons.

Gonzalez-Perez and his colleagues performed an experimental study showing head-on collision between two nerve pulses of less than 5mV undergoing an elastic interaction instead of annihilating upon contact (see [45]). Our model supports their experimental findings without considering adiabatic phenomena associated with the nerve pulse, but through charge reservoirs within the mitochondrial membrane held by a nonlinear capacitor of the cable model.

## Discussion

Intracellular capacitive effects entail self-excitability due to charge ‘soakage’ held by the endogenous membrane capacitor which results in electrotonic signals propagating as solitary waves due to the energy stored in the microstructure [23]. Solitary waves are not solitons since they do not preserve their shape and velocity after collision. Evidence of soliton-like behavior of solitary waves is their elastic interaction after head-on collision between two oppositely directed solitary waves.

The crucial test for solitary waves to be solitons is robustness to collision [42]. Electrical solitons do not undergo nonlinear amplitude modulation during collision because linear superposition is assumed and no phase shift occurs due to a dissipative medium [46]. Based on linear superposition of the elastic interaction, solitary waves were not deformed after head-on collision, preserving their shape and velocity, thus providing support for the solitary waves to be solitons. This result precludes the integrability of the modified nonlinear cable equation, where a phase shift is expected in integrable systems. For instance, Drazin and Johnson [47] define a single soliton solution as a solitary wave (or quasi-soliton), but if more than one soliton appears in the solution then it is called a ‘soliton’. This more stringent definition of a soliton is also referred to as a ‘multi-soliton’ solution or ‘n-soliton’, which requires integrability of the modified nonlinear cable equation. If the modified nonlinear cable equation is nonintegrable then it would imply the absence of n-soliton solutions, but still adhering to the definition of a soliton as a non-dispersing solitary wave, which maintains its shape and velocity after head-on collision [42, 44].

There are mechanical models of soliton propagation in nerve, but none that specifically address electrical solitons. Aizawa and colleagues [48] pointed in the direction that the nerve impulse (i.e., spike or action potential) is a ‘nervous soliton’, but their results did not adhere to the definition mandated of a soliton. The model presented herein constitutes a first attempt at identifying solitons as electrotonic signals propagating in neuronal branchlets with microstructure containing mitochondria. The model differs from that of Poznanski and colleagues [22] where solitonic conduction of electrotonic potentials was due to charged proteins without mitochondrial membranes.

The source term containing charge ‘soakage’ in the cable equation resulted in solitary traveling waves instead of traveling fonts as one would expect in solutions of a cable equation without recovery processes. The inclusion of recovery processes, which is designed to model the slower membrane response of potassium activation and sodium inactivation based on H-H kinetics [49] entails the addition of a ‘recovery’ variable *W* in a subsidiary linear rate equation:

$$\begin{array}{c} \frac{\partial W}{\partial T}=U-W \end{array}$$ (29)

Consequently a spike can be simulated with a refractory period allowing for subsequent spikes to be transmitted, but will result in an inelastic head-on collision upon interaction. Huxley [50] had observed the presence of an unstable subthreshold ‘spike’ midway between electrotonic decay and ignition of a spike. This is not the solitons observed in this paper since the stability or solitonic nature of such subthreshold ‘spikes’ was not evident.

Endogenous electric field is a term used to infer on the absence of any externally applied electric fields. The endogenous electrical field effects are considered to be extracellular fields induced ephaptically, which have been shown to affect spike timing of a neuron [51], but are incapable of triggering or suppressing spike activity in response to synaptic activity [52]. In hindsight, Zhang et al. [53] had concluded that induced extracellular spikes in the absence of synapses and gap junctions observed experimentally must be attributed to the same effects of extracellular fields that was confirmed by computer simulation [54] suggesting a nonsynaptic propagation mechanism consistent with ephaptic field effects. Whether solitonic conduction of electrotonic potentials driven by mitochondrial membrane current within polarized microstructure can induce extracellular spikes would need to be investigated through the inclusion of extracellular potentials (cf. [55])

The implications of a model for understanding the intracellular capacitive effects of macroscopic polarization on membrane potential and on the excitability process in general needs to be developed further. The present formalism can be further extended through inclusion of inhomogeneous linear dissipative neural media of a more general form where the electric conductivity and permittivity are no longer constants, but functions that depend on location and time. In such a circumstance, there is a need to reconsider more realistic cases were inhomogeneities in the conductivity and permittivity is present. One such example is a more general form where the electric conductivity and permittivity are no longer constants, but functions that depend on location and time:

$$\begin{array}{c} D(x,t)=\int \varepsilon (x,s)E(x,t-s)\;ds+P(x,t) \end{array}$$ (30)

and

$$\begin{array}{c} J\left(x,t\right)=\int \sigma \left(x,s\right)E\left(x,t-s\right)\;ds+\frac{\partial D}{\partial t} \end{array}$$ (31)

where *D* is the electric flux density and *J* is the current density. Furthermore, for a nonlinear dissipative neural media, it also requires the susceptibility (*χ*) to be a function of time and location:

$$\begin{array}{c} P\left(x,t\right)=\varepsilon_{0}\int \chi \left(x,s\right)E\left(x,t-s\right)\;ds \end{array}$$ (32)

where *P* is the polarization field in the longitudinal direction (along the cable length). The consequence of this re-evaluation renders the model to include dispersive capacitive effects (i.e., capacitance that is frequency-dependent) seen both theoretically [20] and experimentally [56].

## Conclusion

In this paper, we provided a phenomenological description of the electrolytic microenvironment that assumes electrodiffusion of ions to be reflected by electrically charged homogenous core-conductor, where charge densities are continuous distributions reflecting displacement current in an electrolytic cable with polarized microstructure.

The nonlinear cable equation with a source term representing charge ‘soakage’ in a linearly dissipative medium was derived from Maxwell’s equations under quasi-electrostatic conditions (slow moving electric field) and solved to describe traveling wave solutions as solitary waves. The head-on collision between two oppositely traveling solitary waves produced an elastic interaction confirming the existence of electrical solitons. The charge ‘soakage’ produced non-dispersing effects that sustained the amplitudes of the solitons from dissipating. The results are consistent with solitonic conduction of electrotonic potentials based on spatial and ionic homogeneity, negligible concentration gradients, and extracellular isopotentiality. The effect of polarized microstructure in a cable model with mitochondrial membranes has confirmed that electrotonic signals can be conducted as solitons. In principle, the confirmation of electrotonic signals as electrical solitons complements the 65 year standing H-H model at the subcellular scale.

## Supporting information

S1 Appendix Determination of amplitudes. (PDF)

S2 Appendix Local stability analysis. (PDF)

S3 Appendix Energy dissipation. (PDF)
