# Supplementary material for: Induced mitochondrial membrane potential for modeling solitonic conduction of electrotonic signals
Source: PLoS One. 2017 Sep 7;12(9):e0183677. doi: 10.1371/journal.pone.0183677 (PMC5589106; doi:10.1371/journal.pone.0183677)
Supplement: S1 Appendix — (PDF) [file pone.0183677.s001.pdf]

Induced mitochondrial membrane potential for modeling solitonic conduction of electrotonic signals

R.R. Poznanski[1*], L.A. Cacha[2], J. Ali [2], Z.H. Rizvi[2], P. Yupapin [3,4], S.H. Salleh [5], A. Bandyopadhyay[6]

1 Faculty of Bioscience and Medical Engineering, Universiti Teknologi Malaysia, 81310 Johor Bahru, Malaysia
2 Laser Centre, Ibnu Sina ISIR, Universiti Teknologi Malaysia, 81310 Johor Bahru, Malaysia
3 Computational Optics Research Group (CORG), Ton Duc Thang University, District 7, Ho Chi Minh City, Vietnam
4 Faculty of Electrical & Electronics Engineering, Ton Duc Thang University, District 7, Ho Chi Minh City , Vietnam
5 Centre for Biomedical Engineering, Universiti Teknologi Malaysia, 81310 Johor Bahru, Johor, Malaysia
6 Research Center for Advanced Measurement and Characterization, National Institute for Materials Science, Tsukuba, 305-0047 Japan

*Corresponding Author: poznanski@biomedical.utm.my

Supporting information

S1 Appendix: Determination of Amplitudes

Upon differentiating Eq (26) (in the manuscript) we have the following derivatives

$$\frac{\partial U^*}{\partial T} = 2a_o\upsilon sech^2(X - X_p - \upsilon T)tanh(X - X_p - \upsilon T) = 2\upsilon U^* tanh(X - X_p - \upsilon T)$$
$$\frac{\partial^2 U^*}{\partial X^2} = 4a_o sech^2\left(X - X_p - \upsilon T\right) - 6a_o sech^4\left(X - X_p - \upsilon T\right) = 4U^* - \frac{6U^{*2}}{a_o}$$
$$\frac{\partial^3 U^*}{\partial T\partial X^2} = 8\upsilon U^* tanh(X - X_p - \upsilon T) - \frac{24\upsilon U^{*2} tanh(X - X_p - \upsilon T)}{a_o}$$
$$\frac{\partial U^{*2}}{\partial T} = 4\upsilon U^{*2} tanh\left(X - X_p - \upsilon T\right)$$

Substituting the above derivatives into Eq (15) (in the manuscript) in order to verify that Eq (26) (in the manuscript) is a solution. In case of the nontrivial solution $U^* \neq 0$ the following relations must hold:

$$2\upsilon \tanh(X - X_p - \upsilon T) - 3 + \eta - 8\upsilon\gamma \tanh(X - X_p - \upsilon T) = 0 \quad (1)$$

$$6/a_0 + \frac{24\gamma\upsilon \tanh(X - X_p - \upsilon T)}{a_0} - 8\upsilon \tanh(X - X_p - \upsilon T) - \delta = 0 \quad (2)$$

From Eq (1) we have $tanh(X - X_p - vT) \approx 1$ for $T < (2 - X + X_p)/v$ and substituting this into Eq (2) we have

$$\frac{6}{a_0} + 24\frac{\gamma v}{a_0} \approx 8v + \delta \quad (3)$$

It can be readily shown that Eq(3) holds when $a_0 \approx 6\frac{1+4\gamma v}{8v+\delta}$.
