# Supplementary material for: Induced mitochondrial membrane potential for modeling solitonic conduction of electrotonic signals
Source: PLoS One. 2017 Sep 7;12(9):e0183677. doi: 10.1371/journal.pone.0183677 (PMC5589106; doi:10.1371/journal.pone.0183677)
Supplement: S2 Appendix — (PDF) [file pone.0183677.s002.pdf]

Induced mitochondrial membrane potential for modeling solitonic conduction of electrotonic signals

R.R. Poznanski[1*], L.A. Cacha[2], J. Ali [2], Z.H. Rizvi[2], P. Yupapin [3,4], S.H. Salleh [5], A. Bandyopadhyay[6]

1 Faculty of Bioscience and Medical Engineering, Universiti Teknologi Malaysia, 81310 Johor Bahru, Malaysia
2 Laser Centre, Ibnu Sina ISIR, Universiti Teknologi Malaysia, 81310 Johor Bahru, Malaysia
3 Computational Optics Research Group (CORG), Ton Duc Thang University, District 7, Ho Chi Minh City, Vietnam
4 Faculty of Electrical & Electronics Engineering, Ton Duc Thang University, District 7, Ho Chi Minh City , Vietnam
5 Centre for Biomedical Engineering, Universiti Teknologi Malaysia, 81310 Johor Bahru, Johor, Malaysia
6 Research Center for Advanced Measurement and Characterization, National Institute for Materials Science, Tsukuba, 305-0047 Japan

*Corresponding Author: poznanski@biomedical.utm.my

Supporting information

S2 Appendix: Local Stability Analysis

The equilibrium solution of Eq (15) (in the manuscript) with boundary conditions $U_0(\pm\infty)$=0 is $U_0 = \alpha\kappa V_0$, where $V_o = \frac{a}{b}\text{sech}^2\left[(a\lambda^2 g_a^* r_i)^{\frac{1}{2}}\frac{X}{2}\right]$ [1] and $\lambda = \sqrt{\frac{r_m}{r_i}}$. Linearization of Eq (15) (in the manuscript) yields the linearized version of the nonlinear cable equation:

$$(1+\eta-\delta U_0)U + (1-2U_0)\frac{\partial U}{\partial T} = \frac{\partial^2 U}{\partial X^2} + \gamma\frac{\partial^3 U}{\partial T \partial X^2} \tag{1}$$

Consider the solution of this equation to be written in this form:

$$U(X,T) = \exp(\theta T + ikX) \tag{2}$$

where θ is the eigenvalue of the wave, k is the wave number. Eq (2) is substituted into Eq(1) in order to obtain the dispersion relation:

$$\theta = -\left(\frac{1+\eta-\delta U_0 + k^2}{1-2U_0+\gamma k^2}\right) \tag{3}$$

For local stability $\theta < 0$ and for this dispersion relation given by Eq (3), the equilibrium solution is therefore shown to be locally stable for all $\eta > 0$ and $\gamma > 0$ if the following condition is satisfied:

$$2(1+\eta) < \delta < \frac{2}{\gamma} \tag{4}$$

i.e., the equilibrium potential is

$$V_a = \frac{\eta}{\delta} < 0.5 - \frac{1}{\delta} \tag{5}$$

References

1. Cacha LA, Ali J, Rizvi ZH, Yupapin P, Poznanski RR. Nonsynaptic plasticity model of long-term memory engrams. Journal of Integrative Neuroscience. 2017;16(4):493–509.
