# Supplementary material for: Induced mitochondrial membrane potential for modeling solitonic conduction of electrotonic signals
Source: PLoS One. 2017 Sep 7;12(9):e0183677. doi: 10.1371/journal.pone.0183677 (PMC5589106; doi:10.1371/journal.pone.0183677)
Supplement: S3 Appendix — (PDF) [file pone.0183677.s003.pdf]

Induced mitochondrial membrane potential for modeling solitonic conduction of electrotonic signals

R.R. Poznanski[1*], L.A. Cacha[2], J. Ali [2], Z.H. Rizvi[2], P. Yupapin [3,4], S.H. Salleh [5], A. Bandyopadhyay[6]

1 Faculty of Bioscience and Medical Engineering, Universiti Teknologi Malaysia, 81310 Johor Bahru, Malaysia
2 Laser Centre, Ibnu Sina ISIR, Universiti Teknologi Malaysia, 81310 Johor Bahru, Malaysia
3 Computational Optics Research Group (CORG), Ton Duc Thang University, District 7, Ho Chi Minh City, Vietnam
4 Faculty of Electrical & Electronics Engineering, Ton Duc Thang University, District 7, Ho Chi Minh City , Vietnam
5 Centre for Biomedical Engineering, Universiti Teknologi Malaysia, 81310 Johor Bahru, Johor, Malaysia
6 Research Center for Advanced Measurement and Characterization, National Institute for Materials Science, Tsukuba, 305-0047 Japan

*Corresponding Author: poznanski@biomedical.utm.my

Supporting information

S3 Appendix: Energy Dissipation

Eliminating the nonlinear terms from Eq (14) (in the manuscript) yields the linearized version of the cable equation:

$$V + \frac{\partial V}{\partial T} = \frac{\partial^2 V}{\partial X^2} + \gamma \frac{\partial^3 V}{\partial T \partial X^2} \tag{1}$$

The most elementary wave solution of this equation is the harmonic wave

$$V(X,T) = A \exp[i(kX + \omega T)] \tag{2}$$

where A is the amplitude of the wave,k is the wave number and ω is the angular frequency. In order for $V(X,T)$ given by Eq (2) to be a solution of Eq (1), ω and k must satisfy the relation

$$\omega = i \frac{1 + k^2}{1 + \gamma k^2} \tag{3}$$

A dispersion relation is connected to the phase velocity $v_p = \frac{\omega}{k}$ and the group velocity $v_g = \frac{\partial \omega}{\partial k}$ as long as the problem is non-dissipative, i.e. ω must be real when k is real. For this dispersion relation given by Eq (3), the system is dissipative, so the linearized cable Eq (1) involves energy dissipation. Substituting Eq (3) into Eq (2) gives the

solution:

$$V(X,T) = A\exp[ikX - (\frac{1+k^2}{1+\gamma k^2})T] \tag{4}$$

Accordingly, the exponential decay of Eq (4) is obviously clear for $T \to \infty$.
